# Effects of the Combined Contraceptive Vaginal Ring on Female Sexual Function: A Systematic Review and Meta-analysis

**DOI:** 10.21315/mjms2023.30.1.3

**Published:** 2023-02-28

**Authors:** Sedigheh Abdollahpour, Akram Ashrafizaveh, Elham Azmoude

**Affiliations:** 1 Nursing and Midwifery Care Research Center, Mashhad University of Medical Sciences, Mashhad, Iran; 2 Department of Midwifery, Torbat Heydariyeh University of Medical Sciences, Torbat Heydariyeh, Iran; 3 Department of Midwifery, Neyshabur University of Medical Sciences, Neyshabur, Iran

**Keywords:** contraceptive agents, hormonal, sexual function, vaginal ring

## Abstract

There are controversial debates regarding the effects of contraceptive vaginal ring devices on females’ sexual function. Therefore, the meta-analysis of before-after was conducted on the intervention studies published in the past years to clarify these contradictions. The existing literature on the subject was reviewed by searching through such databases as PubMed, Scopus, ISI Web of Sciences, Embase, Cochrane Library and Google Scholar up to July 2021. Before-after intervention studies that had examined the effect of vaginal rings on females’ sexual function were collected as well. In total, five studies with 369 participants were included in quantitative syntheses. Pooled results from the random-effect model showed that NuvaRing had a positive effect on females’ sexual function three months after insertion (WMD: 2.48; 95% CI: 0.30, 4.67; *P* = 0.026); however, this effect was not significant after 6 months (WMD: 4.38; 95% CI: −4.95, 13.72; *P* = 0.357). Meta-regression analysis suggested that the effect of this device is associated with users’ age and body mass index 3 months after insertion. No publication bias was found by Egger’s test or funnel plots. Overall, the results of this meta-analysis support the view that vaginal ring use is associated with a positive effect on the sexual function of women 3 months after insertion, while the effect of this device on the sexual function of women was insignificant after 6 months. However, given the dearth of available data, it is not possible to reach a definite conclusion on the effect of vaginal rings on females’ sexual function.

## Introduction

Today, several highly effective and safe hormonal contraceptive products are available to control and regulate human fertility behaviour (1). However, possible biological and psychological side effects may occur during the application of these methods which can determine their widespread acceptability. In this regard, it appears that hormonal contraceptives can affect human sexual behaviour (2, 3). Some research suggested that negative changes in sexual function can be a reason for termination and irregular use of contraceptives among women (3, 4). Therefore, sexual problems associated with these methods should be assessed and reviewed regularly before and during the application of different contraceptive methods (5).

There are many studies on the effect of hormonal contraceptives on females’ sexuality. However, since most of these studies have been restricted to oral contraceptives, there is limited evidence in the literature regarding the effect of new combined contraceptive delivery systems on sexuality (2, 4, 6).

Although vaginal rings releasing etonogestrel and ethinyloestradiol are acceptable modern hormonal contraceptives, recent observational studies have shown conflicting results regarding their effect on females’ sexual function (7). Some studies have reported that vaginal rings increased vaginal wetness and sexual satisfaction in women (8–11). In contrast, Gracia et al. (11) revealed a decrement in sexual function 3 months after ring insertion.

Furthermore, negative sexual changes, such as irregular uterine bleeding and physical discomfort during sexual intercourse due to the presence of a foreign body in the vagina attributable to the application of this type of contraceptive can interfere with the females’ sexual function (12, 13). Conversely, psychological reassurance resulting from the elimination of concerns regarding the risk of unintended pregnancy is associated with a positive impact on women’s sexual function (14).

Therefore, given the disagreements in the existing literature, the review of recent evidence on this subject seemed to be necessary. In general, due to the lack of comprehensive data in this area, the current study was conducted to measure the effects of the combined contraceptive vaginal ring on females’ sexual function.

## Methods

### Search Strategy

Databases including PubMed, ISI web of science, Scopus, Embase, Cochrane Library and Google Scholar were searched, up to July 2021, using such search terms as ‘Vaginal Ring’, ‘Nuvaring’, ‘Sexual’, ‘Sexuality’, ‘Sexual Behaviour’, ‘Sexual Dysfunctions’ and ‘Sexual Function’. Moreover, PICO elements were used, including Population, Intervention, Comparison and Outcome. The study population included reproductive-aged women and the intervention consisted of the insertion of a vaginal ring as a hormonal contraceptive method. The vaginal ring is a safe and convenient birth control method and releases 15 μg of ethinylestradiol (EE) and 120 μg of etonogestrel (ENG), daily. The comparison group was the group receiving other contraceptive or no contraceptive method at all. Outcomes consisted of sexual function. This variable was measured using valid tools and reported as mean and standard deviation (SD). Full search terms are available in Supplementary File 1.

Additional relevant studies were also collected by searching through reference lists of all the identified studies.

### Study Selection

Inclusion criteria in the meta-analysis included: i) prospective cohort of intervention studies (randomised controlled trial [RCT], before-after designs or controlled intervention studies) that examined the effect of vaginal ring on sexual function; ii) full articles available in English and iii) sexual function measured in baseline and follow-up and presented as mean ± standard deviation (SD) values or mean difference and SD difference. On the other hand, exclusion criteria included: i) letter or editorial papers, narrative reviews or case reports, and ii) conference abstracts without a full text. No time restriction was applied when searching the relevant studies.

**Figure 1:**
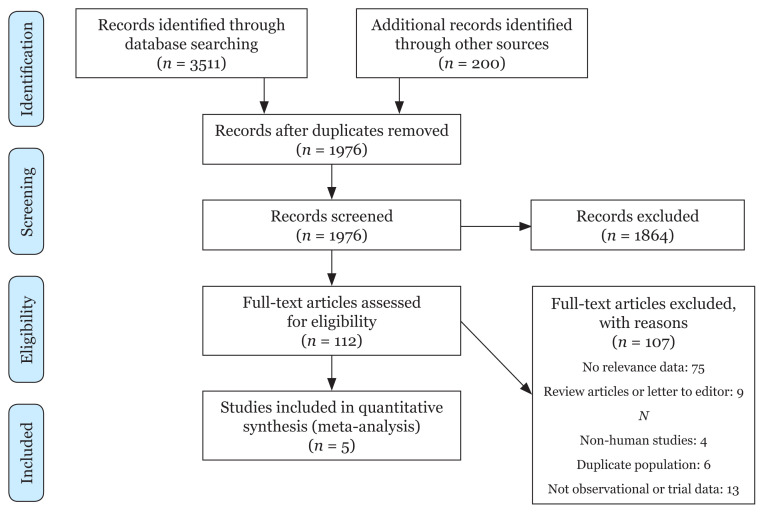
PRISMA flow diagram of the search process

Moreover, study selection was performed independently by two investigators (EA and AA). Any disagreement between the research team members was resolved through discussion. Details of the study selection process are presented in the PRISMA flow diagram in Figure 1.

### Data Extraction and Quality Assessment

All data were reviewed and extracted from the collected studies by two independent authors (EA and AA) using a standardised information sheet. The extracted data included information about the first author, year of publication, study setting, study design (i.e. RCT, before-after study), the follow-up time interval, sample size, sexual function assessment scales, sexual function mean ± SD and age at baseline.

Furthermore, the quality of the included studies in the meta-analysis was evaluated using the National Institutes of Health (NIH) framework for the quality assessment of controlled intervention studies (*n* = 14) and before-after (pre-post) studies with no control group (*n* = 12) through consensus by two authors. The assessed criteria in controlled intervention studies included a description of randomisation, adequate randomisation method, blinding, the similarity of groups at baseline, overall and differential dropout rate, adherence to the study protocols, avoiding other interventions, reliable and valid outcome measurement method, selection of sufficient sample size and intention-to-treat analysis.

Furthermore, 12 sources of bias in the before-after studies with no control group were assessed using a different scale. These included the clarity of study question, description of eligibility criteria and study population, representation of the population, attention to inclusion/exclusion criteria prior to recruitment, selection of proper sample size, description of intervention and outcome measurement, blinding of outcome assessors, follow-up rate, statistical analysis, multiple outcome measures, group-level interventions and individual-level outcome efforts (15). These questions required a yes-no or other responses (i.e. cannot determine, not reported or not applicable). Studies were classified into having a low score (76%–100%), intermediate score (26%–75%) and high risk of bias (0%–25%) (16).

### Statistical Analysis

Effect sizes for the meta-analysis were selected based on the weighted mean difference (WMD) of sexual function score. Subgroup analysis, as well as meta-regression analysis, were performed to determine the source of heterogeneity. Statistical heterogeneity was also measured by the Cochran Q test and the I2 statistic. A funnel plot and Egger’s tests were further used to evaluate publication bias. Sensitivity analyses were also performed to evaluate the effects of each study on the overall results. A random-effect model was used to derive the overall effect. The data synthesis was carried out using Comprehensive Meta-Analysis software (version 2, Biostat, Englewood, NJ, USA). *P*-values less than 0.05 (*P* < 0.05) were considered statistically significant.

## Results

### Study Characteristics

The PRISMA flow chart of included studies is presented in Figure 1. In total, five studies, including 369 women met the predefined inclusion criteria, of which three followed the RCT design and two had a before-after design. It is worth mentioning that four out of these five studies were conducted in Italy and one was performed in the United States. Table 1 summarises the main characteristics of the eligible studies. All studies used a validated tool to assess sexual function using Female Sexual Function Index (FSFI), McCoy Female Sexuality Questionnaire (MFSQ) and Interviewer Ratings of Sexual Function (IRSF). The duration of collected studies ranged from 126 days to 6 months of follow-up.

### Assessment of Quality of Studies

The risk of bias for three studies was rated as moderate, whereas two studies had a low risk of bias. No study met the criteria for a high risk of bias (Table 1).

### Effect of NuvaRing on Sexual Function

**Figure 2:**
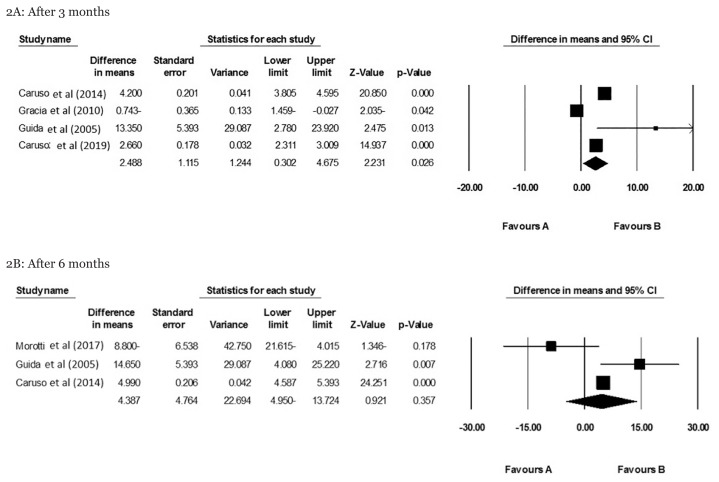
Effect of NuvaRing on sexual function in 3 and 6 months after insertion

Forest plots summarising the meta-analysis of the effect of NuvaRing on the sexual function of women are illustrated in Figures 2A–B. Pooled results from the random-effect model showed that NuvaRing had a remarkable positive effect on the sexual function of women three months after insertion (WMD: 2.48; 95% CI: 0.30, 4.67; *P* = 0.026) (Figure 2A). However, this effect was insignificant after 6 months (WMD: 4.38; 95% CI: −4.95, 13.72; *P* = 0.357) (Figure 2B).

### Heterogeneity, Publication Bias and Sensitivity Analysis of Included Studies on the Effect of NuvaRing on Sexual Function

The Q-test results showed significant heterogeneity among the studies in two time periods (Q-statistic *P* = 0.001, I2 = 97.95% and Q-statistic *P* = 0.022, I2 = 73.87%, respectively).

Meta-regression analysis was also conducted to evaluate the association between sexual function score and potential moderator variables, such as participant’s age and body mass index (BMI). The results suggested that the pooled estimate was associated with the participant’s age 3 months after ring insertion (slope: −0.59; 95% CI: −0.76, −0.42; *P* = 0.001). Furthermore, the results suggested that the effect of ring on sexual function of women varied significantly based on their BMI, 3 months after insertion (slope: −1.56; 95% CI: −2.25, −0.87; *P* = 0.001), though not after 6 months (slope: 0.11; 95% CI: −3.11, 3.33; *P* = 0.945).

**Figure 3:**
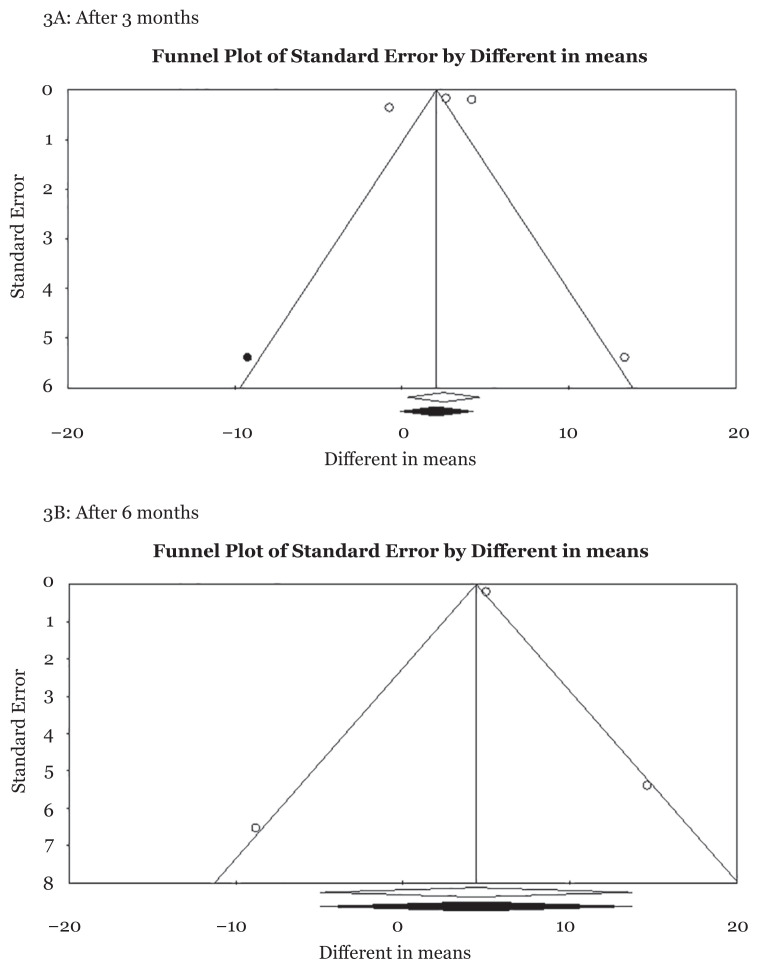
Funnel plot of included studies about the effect of NuvaRing on sexual function

Moreover, a slight asymmetry was observed in Begg’s funnel plot 3 months after ring insertion. No evidence of publication bias was found using Egger’s test (*P* for bias = 0.813, 0.55, respectively) (Figure 3A).

Inconsistently, the funnel plot reflected symmetry in studies conducted on the effect of vaginal rings on females’ sexual function 6 months after insertion. Egger’s test results (*P* = 0.945) showed no publication bias (Figure 3B).

**Figure 4:**
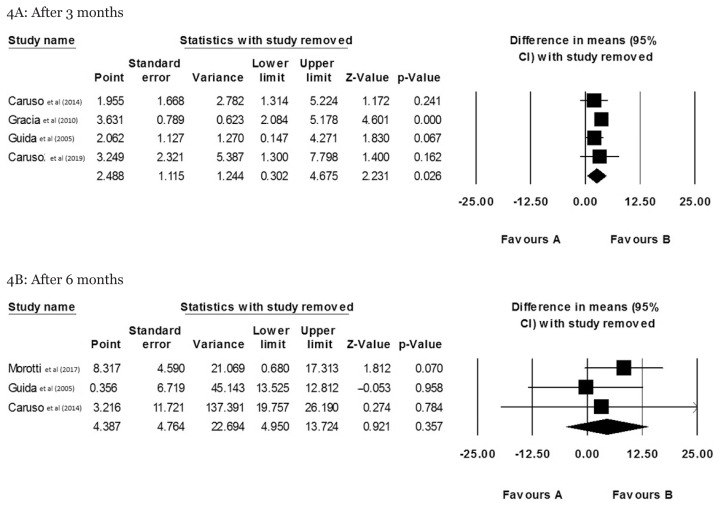
Sensitivity analysis of the NuvaRing effect on sexual function

Additionally, the sensitivity analysis indicated that the exclusion of three studies conducted by Caruso et al. (8), Guida et al. (17) and Caruso et al. (18) could considerably alter the summary effect into non-significant at the third month after insertion (Figure 4A). Inconsistently, no study could change the overall effect into significant in the studies conducted on the effect of vaginal rings on females’ sexual function 6 months after insertion (Figure 4B).

## Discussion

To the best of our knowledge, this is the first study evaluating the effects of vaginal rings on the sexual function of reproductive-age women.

Based on the obtained results, the vaginal ring does not seem to deteriorate sexual functioning in 6 months; although, it has a positive at the third month after insertion. A possible explanation for this finding is that sensation of touching the ring by a partner during intercourse, as a reminder of the contraceptive protection, improved sexual function at the beginning of usage. In this regard, Severy and Spieler (19) declared that penile contact with the ring inside the vagina could be a sexual stimulus for some couples. Furthermore, in one study, there was a significant reduction in sexual activity anxiety among vaginal ring users and their partners, compared to the control group (19). However, this effect seemed to be temporary, and the findings showed that a balance might be established between the advantages and disadvantages of this contraceptive method after 6 months. Moreover, based on evidence, improved lubrication due to local and systemic activity of ethinyl estradiol and more vaginal wetness, as well as improved vaginal flora due to increased number of lactobacilli may improve sexual function in users of these contraceptives (11, 12, 20). Consistently, in a recent study performed by Battaglia et al. (21), increased vaginal wetness was reported among vaginal ring users. In contrast, based on the results of another study, some side effects of this method, such as spotting or other bleeding irregularities, may lead to sexual dysfunction in women (22).

Inconsistent with these results, most previous publications on this area showed either negative effects (12, 22, 23) or an improvement in the sexual domains (17, 18, 21) caused by the application of vaginal ring at the studied period of time (namely, 3 months and 6 months after insertion).

Based on the results of meta-regression analysis, participants’ age and BMI were associated with significant heterogeneity between studies conducted on the effects of vaginal rings 3 months after insertion. However, another study reported an association between sexual comfort and the age of vaginal ring users (13).

Regarding the limitations of this systematic review and meta-analysis, one can refer to the small number of collected studies (low sample size of included studies). Moreover, most of the included studies in the meta-analysis had been carried out in Italy, which makes it impossible to overgeneralise the results obtained in the present study. Moreover, given that the overall risk of bias for all included studies was moderate, more high-quality RCTs are needed to clarify the precise effects of vaginal rings on the sexual function of women.

## Conclusion

Based on the obtained results in this meta-analysis, it can be concluded that the application of vaginal rings is associated with a positive effect on the sexual function of women 3 months after insertion. However, this device had no impact on the sexual function of women after 6 months. Given the limited available data on the effect of vaginal rings on the sexual function of women, future randomised, controlled and double-blinded studies with low risk of bias levels are recommended to increase the validity of the results obtained in this study.

## Supplementary Information

**Supplementary File 1:** Search strings and results presented by database

Database	Number of publications
Embase.com	641
Cochrane	126
Web-of-science	21
Scopus	586
Pubmed	1987
Google Scholar	150

Total	3511

Electronic databases searched till the 12th of July 2021 Search strings	
*Embase.com*	
((Vaginal Ring OR Nuvaring):ab,ti) AND ((sex OR sexuality OR ‘sexual arousal’ OR ‘sexual dysfunction’/exp OR ‘psychosexual disorder’ OR ‘sexual function’ OR ‘Female Sexual Function Index’ OR (sexual* OR arousal* OR anorgas* OR orgas* OR libido* OR dyspareunia):ab,ti)	
*Cochrane*	
((Vaginal Ring OR Nuvaring):ab,ti) AND ((sexual* OR arousal OR anorgas* OR orgas* OR libido OR dyspareunia OR desire OR lubrication):ab,ti)	
*Web-of-science*	
TI = ((vaginal ring OR Nuvaring) AND (sexual* OR arousal OR anorgasm* OR orgasm* OR libido OR dyspareunia OR desire OR lubrication))	
*Scopus*	
TITLE-ABS-KEY((Vaginal Ring OR Nuvaring) AND (sexual* OR arousal OR anorgas* OR orgas* OR libido OR dyspareunia OR desire OR lubrication))	
*Pubmed*	
(Vaginal Ring [Mesh] OR Vaginal Ring [ TIAB] OR Nuvaring [TIAB] OR Ring, Vaginal[Mesh]) AND (sexual*[TIAB] OR Sexual and Gender Disorders[Mesh] OR Sexuality[Mesh] OR Sexual Behavior[Mesh] OR Sexual Dysfunction, Physiological[Mesh] OR Sexual Dysfunctions, Psychological[Mesh] OR arousal[Mesh] OR arousal[TIAB] OR orgasm[Mesh] OR orgas*[TIAB] OR libido[TIAB] OR libido[Mesh] OR dyspareunia[TIAB] OR dyspareunia[Mesh] OR desire[TIAB] OR lubrication[Mesh] OR ubrication[TIAB])	
*Google Scholar*	
(Vaginal Ring OR Nuvaring) AND (sexual OR sexuality OR arousal OR orgasm OR libido OR dyspareunia OR desire OR lubrication OR libido)	

## Figures and Tables

**Table 1:** Characteristics of included studies

First author	Country/Year	Study design	Sample size	Age (age ranges)	Scale	Follow-up period	Main results	Risk of bias
Caruso	Italy/2014	Before-After intervention study	52	23.8 ± 3.8 (18–32)	FSFI	126 days–134 days	Improved sexual function	Moderate
Caruso	Italy/2019	Randomised trial, Parallel Control group: Another type of vaginal ring	46	26.9 (18–37)	FSFI	3 and 6 months	Improved sexual function	Moderate
Gracia	USA/2010	Randomised trial, Parallel Control group: contraceptive patch	226	26.17 ± 5.50	FSFI	3 months	Decrease sexual function	Moderate
Guida	Italy/2005	Randomised, controlled, study, parallel Control group: oral contraceptive	26	30.6 ± 2.4 (22–34)	IRSF	3 and 6 months	Improved sexual function	Low
Morotti	Italy/2017	Before-After intervention study	19	27.1 ± 3.4 (18–35)	MFSQ	3 months	Decrease in one subscale	Low
